# Overdenture pickup without retaining material; a novel dental technique

**DOI:** 10.3389/fdmed.2025.1589993

**Published:** 2025-06-11

**Authors:** Noha Waleed Barakat, Mahmoud Mokhtar El Far, Ahmed Mustafa Hashim Kotb

**Affiliations:** Department of Prosthodontics, Faculty of Dentistry, Cairo University, Giza, Egypt

**Keywords:** overdenture, pickup, mandibular overdenture, ExoCAD, digital denture

## Abstract

This article describes a novel technique for overdenture metal housing pickup for a digitally fabricated overdenture using an extraoral scanner and ExoCAD software. This technique could help the clinician save time and reduce the hassle of extra visits and additional laboratory steps.

## Introduction

It has been more than 15 years since the landmark McGill Consensus Statement that described “mandibular two-implant overdenture as the first-choice standard of care for edentulous patients”. It has also been reported that implant-retained overdentures have superior performance, and improve patient satisfaction and quality of life compared to conventional dentures (1).

Blending the attachment system with the conventional denture in terms of “overdenture” has become increasingly used nowadays. One of the most popular attachment systems is the Locator attachment as it could be used in patients who have limited interarch distance due to their low profile, They are also resilient, self-aligned, compensate for implant malalignment up to 20 degrees, and are considered advantageous when retrofitting an existing old denture (2, 3).

However, one of the most common prosthetic complications is the detachment of metal housing from the undersurface of the denture base, in other words, failure of the pickup procedure (3–5). This may affect the clinical performance of the overdenture until proper care is provided.

The rapid development of CAD-CAM technology has provided clinicians with more options in designing and milling different dental components and prosthesis. From this perspective, the authors implemented the recent CAD CAM applications in an attempt to solve the problem of metal housing detachment, by introducing a novel pickup technique for metallic housings of locator attachments in digitally-fabricated overdentures.

## Technique

**Figure 1:**
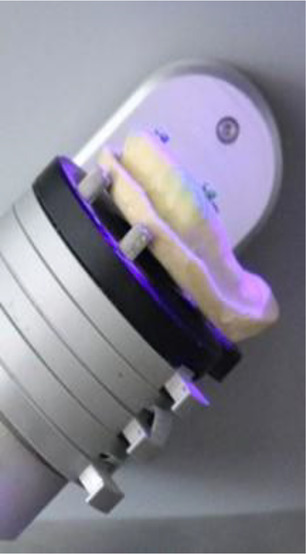
Model scanning procedure.

**Figure 2:**
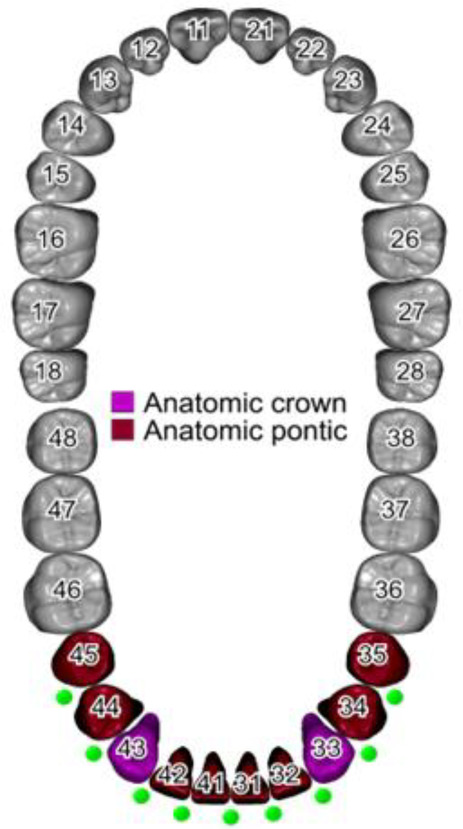
Screenshot of the initial parameters used in the designing software.

**Figure 3:**
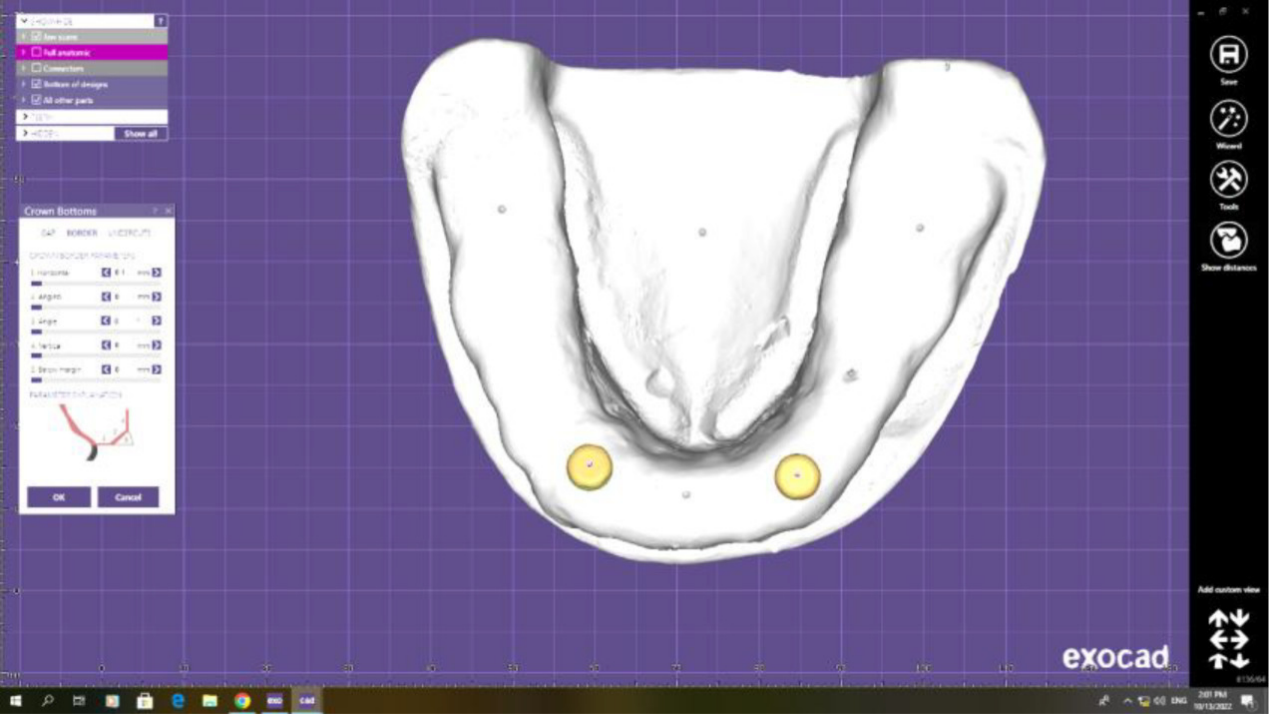
Screenshot of designing software interface for cement gap parameter adjustment to be zero.

**Figure 4:**
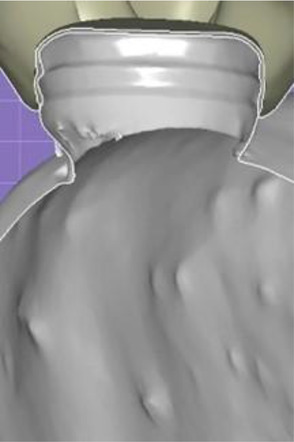
Screenshot of designing software interface showing cross-section of zero gap distance between the locator metal housing and the fitting surface of the prosthesis.

**Figure 5:**
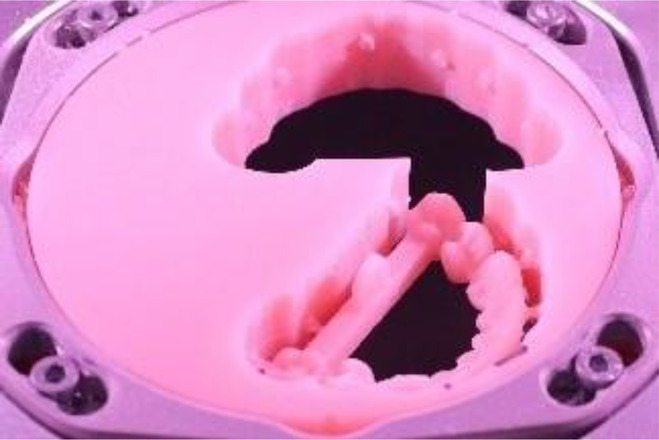
Milling procedure of the superstructure.

**Figure 6:**
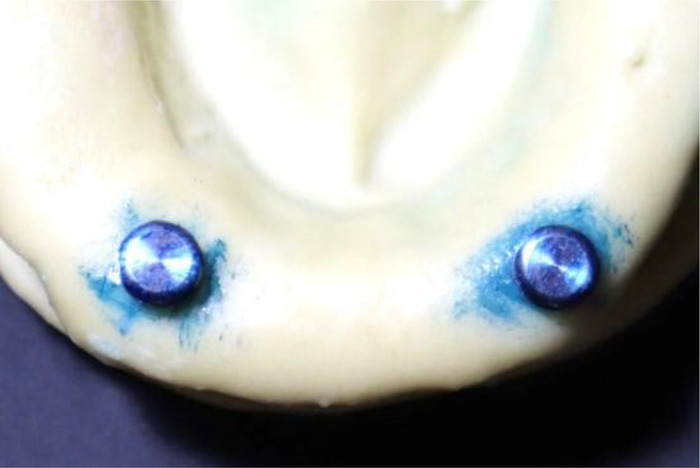
Checking any interference by using a liquid marker.

**Figure 7:**
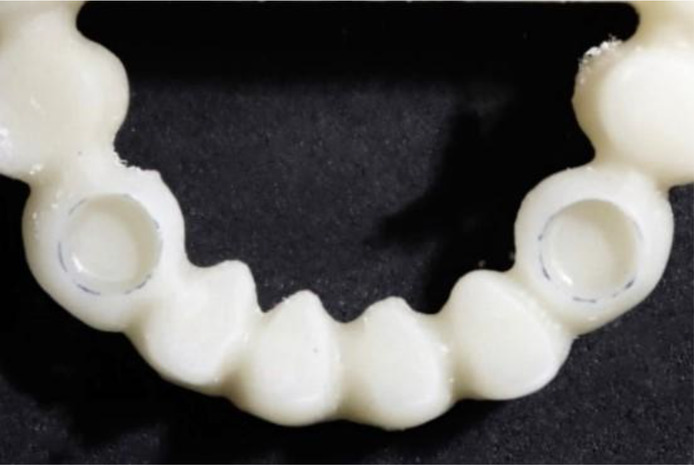
Fine blue line of interference marking after the liquid marker was applied.

1. Prepare a mandibular model with two implants and their locator attachments with their metallic housings (NEOBIOTECH implant system) then scan them and the area of the edentulous ridge with the extraoral laboratory scanner (IDENTICO company) to be transferred into a digital file in the form of an STL file (Figure 1).
2. Import the digital STL file of the scan to ExoCAD dental designing software (VERSION 2.2 VALLETTA) to design the overdenture simulator prosthetic part.
3. Choose the job order to design copings on the Locator metal housings position (Figure 2).
4. Use the automatic surveying tool to define the long axes of both metal housings and block out the undesirable undercuts by the software.
5. Adjust the design parameters to be with zero µm gap distance between the metal housing axial walls and the fitting surfaces of the copings (Figures 3, 4).
6. Wax up the artificial teeth virtually on top of these copings and continue to complete the rest of the overdenture design.
7. Export the completed design into an STL file to be ready for milling.
8. Separate the milled prosthesis from the PMMA puck (YAMAHACHI DENTAL MFG company) by using a carbide bur and straight handpiece (Figure 5).
9. Apply a thin layer of a liquid marker (Ceramill marker BLU, AMANN GIRRBACH company) on the external surface of the metal housings by using a brush (Figure 6).
10. Try to seat the prosthesis (overdenture) in its position on the model. A fine blue line may appear on the prosthesis where it interferes with the metal housing during the seating attempt (Figure 7).
11. Finish this fine line with a finishing bur and finally remove the metal housings from the model and secure them in their positions within the pick-up cavities in the fitting surface of the prosthesis using finger pressure.

## Discussion

The hybrid structures between polymer and metal have effectively produced a lighter well-functioning dental prosthesis. Currently, this heterogenicity faces the limitations of traditional joining methods. The traditional techniques to join dissimilar materials -polymeric and metallic- parts depend on mechanical interlocking and adhesive bonding. The use of adhesives promotes continuous contact between the parts’ surfaces (6).

However, the use of adhesives needs surface treatment or specific surface qualities depending on the type of adhesive material to be used which adds costs and working time. In addition, this adhesive interface is more prone to be debonded or fractured. That's because this joint is considered a weak interface (6, 7).

Other physical retention methods used in the dental field, intimate contact and friction of structures, were found to be a very common phenomenon applied in removable and fixed prostheses. In other words, if surfaces of two different bodies were in contact and parallel to each other, they would generate what is so-called the joint area. The present dental technique applied this previous rule, and used, frictional force, and intimate contact to achieve the actual contact area between the external surface of the Locator metal housing axial surfaces and the internal surface within the denture pickup cavity (8).

**Figure 8:**
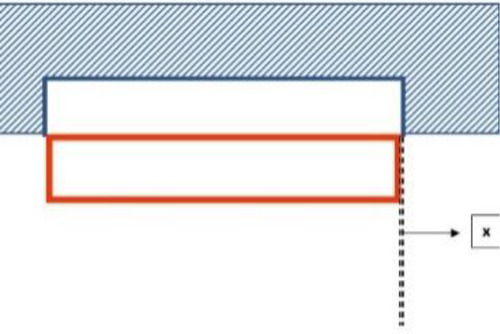
Showing the close-fit with *x* distance = almost zero.

In the same context, there are three types of engineering fit: transition (close) fit, interference fit, and clearance fit, which are distinguished among mechanical joints. Close fit which is applied in our present dental technique is defined as the application of considerable force to connect both elements, so, that parts that are fitted get wedged and become hardly separable (Figure 8). This close fit can be achieved in case of the absence of a gap between the internal surface of the denture pickup cavity and the external surface of the Locator metal housing where they are in direct contact. So, when an occlusal load is applied, no deformation is allowed to destroy the retention force (friction) between the two surfaces by shear or sliding (8, 9).

On the other hand, the advent of the digital dentistry era has enabled the application of techniques that were previously unattainable. For instance, in conventional workflows, it was not possible to precisely control the gap distance between the Locator metal housing and the denture base. However, due to the accuracy and customizable parameters available in CAD software, it is now feasible to achieve a zero-gap fit, resulting in an optimized and intimate adaptation between components. This advancement introduces a need to discuss the digital denture design workflow and the specific modifications implemented in this study, which differ from the conventional clinical protocol due to the *in vitro* nature of the experimental design.

In a typical clinical scenario, the operator would scan the Locator abutment with its metal housing in place to ensure accurate positioning. Consequently, the CAD designer would follow a similar workflow to that described in the methods section, with two exceptions. First, the bar-like structure included in our design for checking the retention of the pickup technique would not be incorporated in a clinical design. Second, in patient-based applications, it would be necessary to add a gingival portion beneath the teeth using the virtual gingiva design option in the software, which simulates the soft tissue contours and supports prosthetic esthetics and function.

From the biomechanical point of view, saliva may play a role in frictional retention and hydraulic adhesion enhancement (8, 9). This implies that studying the intricate surface chemistry at the micron-scale level—particularly the interactions occurring between the lubricant (saliva) and the other two joint surfaces could provide valuable insights into denture attachment performance.

Saliva functions not only as a lubricant but also as a medium that modulates interfacial surface tension, viscosity, and wetting behavior, all of which can influence the retention characteristics of overdenture attachments such as Locators. Furthermore, investigating the influence of the liquid medium in creating and maintaining a tight air-seal within the narrow gap that exists between the denture base and the Locator metal housing represents an additional and promising avenue for future research exploration on clinical cases to evaluate the retentive forces of milled overdenture without using a retaining material during pickup technique.

The capillary and viscous forces generated by saliva within these confined interfaces may contribute to retention through hydraulic adhesion mechanisms, as supported by the principles of fluid dynamics in prosthodontic applications (10). Additionally, recent advancements in tribology and nanotribology suggest that bio-lubrication phenomena at the micro- and nano-scale can significantly alter interfacial behavior, warranting further exploration of saliva's rheological properties and their effect on attachment system efficacy (11, 12). Understanding these mechanisms more deeply could contribute to optimizing attachment design, improving long-term prosthesis stability, and enhancing patient comfort and satisfaction.

## Summary

A novel technique for the Locator overdenture pickup is explained. The technique can potentially solve one of the frequent clinical problems facing the implant overdenture prosthesis. It's -yet- going to be tested clinically and compared it with the traditional methods.

## Data Availability

The original contributions presented in the study are included in the article/Supplementary Material, further inquiries can be directed to the corresponding author.
